# Modulation of Electromagnetic Damping and Charge–Spin Conversion in Pt/Py_100−x_Gd_x_ Heterostructure

**DOI:** 10.3390/ma19122601

**Published:** 2026-06-17

**Authors:** Hongzhan Ju, Jinxiang Wu, Xiaotian Zhao, Long Liu, Wei Liu

**Affiliations:** 1 School of Electronics and Integrated Circuits, Aerospace Information Technology University, Jinan 250299, China; juhongzhan@aitech.edu.cn; 2 Shenyang National Laboratory for Materials Science, Institute of Metal Research, Chinese Academy of Sciences, Shenyang 110016, China; jxwu18s@mail.ustc.edu.cn (J.W.); wliu@imr.ac.cn (W.L.)

**Keywords:** spin nano-oscillators, spin–orbit torque, permalloy-based alloy, magnetic damping, spin Hall angle, magnetic heterostructure

## Abstract

**Highlights:**

- Gd doped in Py fabricated by magnetron sputtering and magnetic properties in Pt/Py-Gd films.
- The magnetic damping coefficient is significantly enhanced by Gd doping, which will be beneficial for fast-switching spintronic devices.
- The Gd-Py alloy reveals obvious better control of the ST-FMR linewidth.
- The spin Hall angle of the Pt/Py-Gd system is largely improved compared with that of the Pt/Py system.

**Abstract:**

Permalloy (Py) is a crucial component in spin nano-oscillators due to its excellent soft magnetic properties. Due to orbital angular momentum quenching, Py exhibits very low magnetic damping. It reduces intrinsic energy dissipation during precession, which is beneficial for lowering operational power consumption and enhancing the thermal stability of certain memory devices. But lower magnetic damping limits its application in fast-switching spintronic devices. Thus, in this work, the rare earth element Gd is introduced into Py to further enhance the spintronic performance of Py_100−x_Gd_x_ alloys. Through spin-torque ferromagnetic resonance measurements (ST-FMRs), the maximum spin Hall angle of the system was calculated to be 0.149 when x = 20, significantly exceeding that of 0.042 in the pure Py sample. Additionally, Gd doping significantly enhances the ability to modulate the magnitude of the linewidth. Also, as the Gd content in the alloy increased, the magnetic damping coefficient of the device gradually rose, reaching a peak in the sample with 17% Gd content. The maximum magnetic damping coefficient of the Py-Gd alloy was 0.051, representing an approximate 2.4-fold increase compared to that of pure Py. The findings of this study confirm that the use of rare-earth elements is highly effective in tuning the performance of spintronic devices and provide support for the development of highly efficient SOT devices. It is noted that the regulation of magnetic damping by Py-Gd holds significant implications for enhancing the magnetization switching speed of SOT devices and reducing the drive current density for microwave emission in spin nano-oscillators.

## 1. Introduction

Spin nano-oscillators are novel spintronic devices based on magnetoresistance effects and spin-transfer torque (STT). It relies on the precession of magnetic moments in nanoscale magnetic structures to generate high-frequency microwave signals (typically in the GHz to THz range). Characterized by their small size, fast frequency tunability, and extremely low power consumption, they hold significant development potential in areas such as terahertz communications, high-performance radars, and brain-inspired computing [1,2,3]. Today, high-speed read/write capabilities have always been a core requirement in various applications of spintronic devices, necessitating magnetic materials with efficient dynamic response [4,5]. Magnetic damping is a key parameter controlling the speed of magnetization switching in spin nano-oscillators [6]. Permalloy (Py) is one of the most critical materials based on low coercivity and high permeability in the research of spin nano-oscillators and other spintronic devices. Py exhibits very low magnetic damping influenced by orbital angular momentum quenching, which is beneficial for STT-MRAM, with lower intrinsic energy dissipation during precession. However, the lower magnetic damping significantly limits its application in spintronic devices requiring fast switching. According to previous studies, doping 3d ferromagnetic metals with rare-earth elements can effectively increase the magnitude of damping [7,8,9]. It is well known that rare-earth elements exhibit ferromagnetic or antiferromagnetic interactions with transition metal elements depending on the filling state of their 4f electron shells. The magnetic properties of rare-earth-transition-metal systems can be effectively tuned by altering the composition or temperature. On the other hand, Py is also one of the most popular magnetic materials in spin-orbit torque (SOT), a method of switching magnetic moments that originated from the spin Hall effect and Rashba effect [10,11]. In recent years, researchers have discovered a spin-splitting effect in RuO_2_/Py heterostructures, laying a solid foundation for spin devices based on alter-magnets [12]. SOT is the theoretical basis of SOT-MRAM, which is a promising magnetic storage technology. However, compared to STT, traditional SOT devices require an in-plane magnetic field to assist magnetization switching, which poses a significant challenge for the application of SOT-MRAM [13,14,15]. Currently, the mechanisms for field-free SOT magnetization switching have become relatively mature with the use of various methods [16,17,18,19,20,21]. With the application of field-free switching technology, the major semiconductor foundries like TSMC have acquired the capability to produce SOT-MRAM [22,23,24]. Therefore, exploring materials with high charge-to-spin conversion efficiency is still crucial to further advance the commercialization of SOT devices [25]. In addition, the alloys play a critical role in the research of SOT devices, which can enhance the spin Hall angle, reduce device energy consumption, and even enable field-free switching [26,27,28,29]. In studies focusing on improving the spin Hall angle, the alloy is predominantly placed within the heavy metal layer (HM). Using alloys to improve the charge-to-spin conversion efficiency in magnetic layers (FM) has not yet received sufficient attention. In recent years, SOT devices based on heavy rare-earth elements have garnered widespread interest as well. Researchers have achieved multi-state SOT switching, field-free switching, and enhanced SOT efficiency in devices doped with heavy rare-earth elements like Ho, Tb, and Gd, respectively [30,31,32,33]. Among them, Gd plays a significant role in improving SOT device efficiency with a single 5d electron [34]. For instance, using the PtGd alloy as the heavy metal layer can effectively increase the spin Hall angle [35]. The antiferromagnetically coupled Co/Gd multilayers can effectively suppress spin dephasing in the magnetic layer, thereby significantly enhancing the SOT efficiency [36]. Furthermore, the combination of Permalloy with heavy rare-earth elements also helps improve the spin Hall angle of devices. It is found that the spin transmissivity at the antiferromagnetically coupled Py/Ho interface is greater than that at Py/Pt and Py/Pd interfaces [37]. Based on this, this work focuses on doping Py with the rare-earth element Gd. The magnetic damping coefficient and SOT performance of the Pt/Py_100−x_Gd_x_ system are systematically investigated using the spin-torque ferromagnetic resonance (ST-FMR) technique. As the impact of Gd doping on the magnetic damping of Py-Gd alloys has not been clearly established [7,38,39], this work helps elucidate the relationship between magnetic damping and rare-earth doping content in Py-Gd alloys. Thus, this work provides effective theoretical support for designing rare-earth-based spin devices with high spin Hall angles and faster switching speeds.

## 2. Experimental Methods

**Figure 1:**
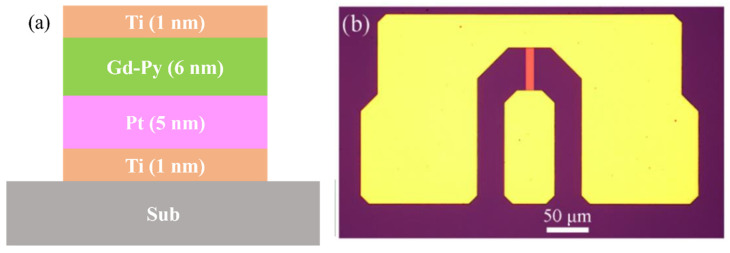
(**a**) The illustration of Pt/Py_100−x_Gd_x_ magnetic multilayers. (**b**) Optical image of a microstrip device for ST-FMR measurements.

Figure 1a shows the magnetic multilayers consisting of Ti (1)/Pt (5)/ Py_100−x_Gd_x_ (6)/Ti (1) (thickness in nm). All samples in this work were fabricated on a thermalized SiO_2_ substrate at room temperature with a high-vacuum DC NanoTorr-100 magnetron sputtering system (Micro Magnetics, Inc., Fall River, MA, USA). In the stacks mentioned above, Ti worked as a buffer and cover layer, and Pt worked as an HM layer to provide spin current. The Ti cover layer effectively blocks oxygen diffusion downward into the Py-Gd layer when the sample is exposed to an ambient atmosphere. To clarify, the Py_100−x_Gd_x_ alloy worked as the FM layer, and x represented the nominal doping ratio of Gd in the Py-Gd alloy (in atomic percent), ranging from 0 to 20. Also, Py in this work refers to Ni_80_Fe_20_. The compositional variation of Py_100−x_Gd_x_ was achieved by co-sputtering. During co-sputtering, the deposition rate of Gd was kept constant at 0.19 Å/s. The doping ratio in the alloy layer was adjusted by varying the sputtering power of Py, corresponding to a deposition rate range of 0.25 Å/s to 1.19 Å/s. The deposition rates for the Ti and Pt targets were kept at 0.68 Å/s and 0.40 Å/s, respectively. It is noted that Gd is highly reactive and readily oxidizes. Thus, a pre-sputtering step was also carried out to eliminate Gd-based oxide layers on the surface of the Gd target. All deposition rates were settled by the weighting method using an analytical balance. The base pressure of the chamber was better than 4 × 10^−7^ Torr prior to sputtering. High-purity Argon (Ar) was used as the working gas, and the pressure was maintained at 2 mTorr during deposition. All targets were pre-sputtered to remove surface oxide layers before formal deposition. In addition, to satisfy ST-FMR measurements, a 50 μm × 10 μm rectangular microstrip device was fabricated by the lift-off method. The shape of the device was shown in Figure 1b. Then, Ti (50 nm)/Cu (500 nm) G-S-G style coplanar waveguide (CPW) electrode pads, fabricated by the lift-off method, were deposited by magnetron sputtering as well to provide electrical contact. ST-FMR tests were carried out on self-established high-frequency detection platforms. During the experiment, a frequency-modulated radio frequency (RF) current generated by an SMB 100A was injected into the microstrip device. Through the rectification effect of anisotropic magnetoresistance (AMR), a voltage signal was generated across the device and measured by an OE1022 lock-in amplifier (Sine Scientific Instrument, Guangzhou, China). An external magnetic field was applied in the plane of the sample. This field could be oriented arbitrarily from 0° to 360° via a motorized rotation stage, allowing the measurement of the ST-FMR signal as a function of the magnetic field’s magnitude, angle, and the RF. Furthermore, this system was also able to measure the AMR of the sample. The static magnetic properties of the samples were measured using a vibrating sample magnetometer (VSM, produced by Eastchanging, Beijing, China). All measurements described above were performed at room temperature.

## 3. Results and Discussion

### 3.1. Magnetic Properties

**Figure 2:**
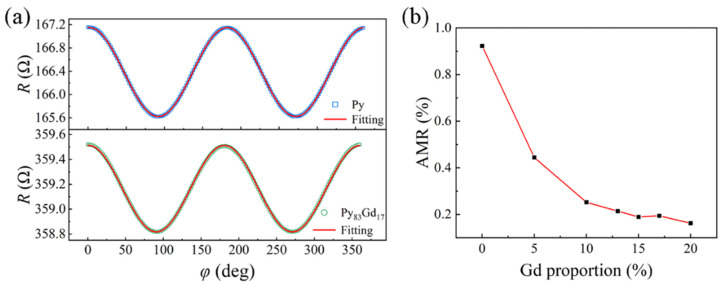
(**a**) The magnetoresistance *R* as a function of the angle *φ* between the magnetic field and the current for the two samples with x = 0 and 17. (**b**) The dependence curve of AMR on Gd content.

Firstly, the magnetic properties of Pt/Py_100−x_Gd_x_ multilayers were studied. Since the ST-FMR signal arises from the rectification effect of the AMR in the sample, we first measured the magnitude of the AMR for each sample [40]. Figure 2a shows the angular dependence of the magnetoresistance of the Py and Py_83_Gd_17_ samples under an in-plane rotating magnetic field of 300 mT. Here, *φ* = 0° corresponds to the magnetic field being parallel to the current direction, where the maximum value of anisotropic magnetoresistance was achieved. As the field angle varies, the magnetoresistance R follows a *cos*^2^*φ* functional relationship, as indicated by the red fitting curves in the figure, which is consistent with the description of AMR given by Equation (1) [41].

(1) $$R=R_{0}+\Delta R{cos}^{2}\varphi ;$$

In Figure 2b, the coefficient of AMR was calculated using $\Delta R/R_{0}\times 100\%$. It is noted that the coefficient of AMR is around 0.92% in the pure Py sample. As the Gd content increases, the AMR effect in the device decreases significantly. The decrease in AMR may cause easier magnetization switching in SOT devices and further lower the critical switch current. When the Gd content reaches 20%, the AMR coefficient is 0.16%. This phenomenon can be attributed to the gradual destruction of the Py crystal structure by the doping of Gd, which reduces its ordering and consequently enhances random scattering, leading to a reduction in the AMR coefficient [38].

**Figure 3:**
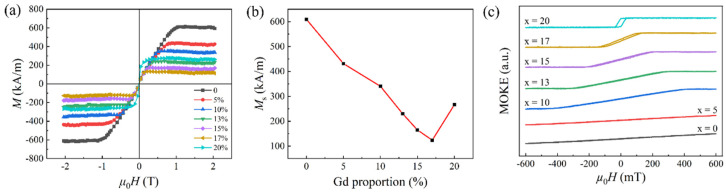
Magnetic properties of the samples. (**a**) M-H curves in the out-of-plane direction for the samples with different Gd contents. (**b**) The variation in saturation magnetization *M_s_* with the Gd content. (**c**) MOKE loops of samples with different Gd contents.

Figure 3a presents the M-H curves of the samples in the out-of-plane direction and the corresponding variation in saturation magnetization (*M_s_*) with Gd content. It is clear that all samples exhibit very small coercivity, indicating excellent soft magnetic properties. In the samples with x ≤ 17, the M–H curve exhibits pronounced hard-axis characteristics. But in the sample with 20% Gd, the M-H curve shows a trend in out-of-plane characteristics. As the Gd content increases, the *M_s_* of the samples gradually decreases, which can be attributed to the antiferromagnetic coupling between Gd and Py in the rare-earth-transition-metal (RE-TM) alloy. This phenomenon leads to the cancellation of a significant portion of the magnetic moments. Moreover, due to the formation of this RE-TM alloy with strong bulk anisotropy, the perpendicular magnetic anisotropy of the samples is also enhanced, manifested as a continuous decrease in the magnetic operations required for out-of-plane magnetization with increasing Gd content, as shown in the M-H curves. When the Gd content reaches 20%, the M-H curve in the out-of-plane direction exhibits some easy-axis characteristics, and the *M_s_* of the sample also increases, as shown in Figure 3b. This phenomenon is the sign of reaching the magnetic compensation point in the sample with x = 17. In summary, the material exhibits a notable transition in magnetic anisotropy, with the easy magnetization axis gradually shifting from the in-plane to out-of-plane direction near the compensation point. This behavior is closely related to the inhomogeneous magnetic moment distribution induced by Gd doping. As the Gd concentration increases, the antiferromagnetic coupling between the rare-earth and transition-metal sublattices varies, resulting in a decreased *M_s_* near the compensation point. This promotes the emergence of out-of-plane magnetization characteristics. The changes in magnetic anisotropy can also be elucidated from the magneto-optical Kerr effect (MOKE) hysteresis loops of the samples.

In Figure 3c, the hysteresis loop still cannot be saturated under an out-of-plane field of 600 mT when the Gd content in Py is no more than 5%. The loop appears as a sloped-like line over the entire field range. In addition, the slope of the MOKE signal versus the external field is also similar. As the Gd content increases, accompanied by the appearance of slight magnetic hysteresis, the MOKE loops begin to show signs of saturation. When *x* = 20, this region has developed into a parallelogram-like shape. Moreover, as shown in Figure 3c, all samples exhibit the same switching polarity in their MOKE loops.

### 3.2. The ST-FMR Analysis

**Figure 4:**
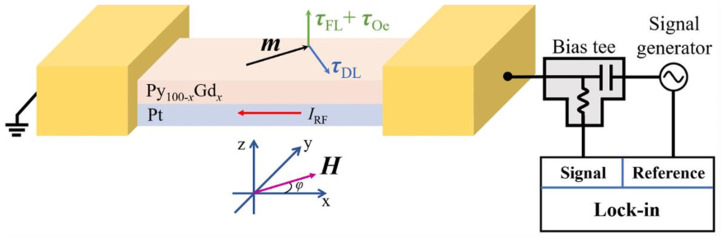
The illustration of ST-FMR measurements.

Then, the ST-FMR tests were carried out on samples, and Figure 4 shows the illustration of ST-FMR measurements. When an RF current is injected into the microstrip device in Figure 1b, the magnetization of the sample undergoes periodic precession under the combined action of the current-induced spin–orbit torques and Oersted field torque. The spin–orbit torques include damping-like torques and field-like torques. The damping-like torque and the field-like torque are proportional to m×σ×m and m×σ, respectively, where m and σ are the unit magnetization vector in the ferromagnetic layer and the unit polarization vector of the spin current, respectively [13,14,15]. Also, these torques will yield a damping-like SOT field (*H_DL_*) and a field-like SOT field (*H_FL_*), respectively [13,14,15]. This, in turn, causes oscillatory variations in the AMR of the sample. Since the frequency of the current matches the period of the AMR variation, a DC voltage signal *V_mix_* can be extracted across the device via the rectification effect, which constitutes the ST-FMR signal. When an external magnetic field is applied in the plane of the sample, resonance occurs in the magnetization precession process whenever the RF and the magnitude of the applied field satisfy the Kittel equation [42,43]. In this work, the frequency of the RF current was fixed, and the Kittel equation was satisfied by scanning the external magnetic field. Therefore, the ST-FMR signal can be expressed as the sum of a symmetric Lorentzian function and an anti-symmetric Lorentzian function [42,44,45]. The *V_mix_* can be expressed as Equation (2).

(2) $$V_{mix}=V_{S}\frac{{\left(\mu_{0}\Delta H\right)}^{2}}{{\left(\mu_{0}H-\mu_{0}H_{0}\right)}^{2}+{\left(\mu_{0}\Delta H\right)}^{2}}+V_{A}\frac{\left(\mu_{0}H-\mu_{0}H_{0}\right)\mu_{0}\Delta H}{{\left(\mu_{0}H-\mu_{0}H_{0}\right)}^{2}+{\left(\mu_{0}\Delta H\right)}^{2}};$$

In Equation (2), *μ*_0_*H*_0_ represents the magnitude of the applied magnetic field at which resonance occurs, hence referred to as the resonance field, and *μ*_0_Δ*H* is the linewidth of the resonance peak, corresponding to half of the half-width at half-maximum of either the symmetric or anti-symmetric Lorentzian function. The first term on the right-hand side of the equation represents the symmetric Lorentzian function component, where *V_S_* is its amplitude, and it is proportional to the damping-like effective field. The second term on the right-hand side corresponds to the anti-symmetric Lorentzian function component, for which its *V_A_* amplitude is proportional to the sum of the field-like effective field and the current Oersted field.

**Figure 5:**
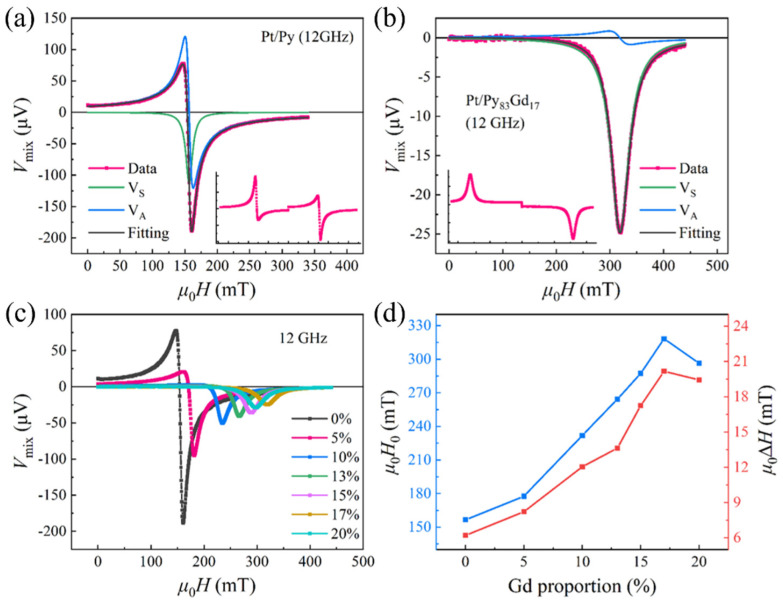
Measurement results of ST-FMR. (**a**,**b**) ST-FMR spectra and the corresponding fitted curves in the sample with (**a**) Py and (**b**) Py_83_Gd_17_, with the field oriented at *φ* = 45°. Inset: ST-FMR spectra for both positive and negative fields. (**c**) ST-FMR spectra for samples of different Gd content at *φ* = 45° and (**d**) variations of *μ*_0_*H*_0_ and *μ*_0_Δ*H* with the Gd content.

Figure 5a,b show the variation in the rectified voltage signal across the device for the Py and Py_83_Gd_17_ samples, respectively, under a 12 GHz radio-frequency current while sweeping the magnetic field along the *φ* = 45° direction. Fitting the data using Equation (2), as indicated by the solid black lines in the figures, yields the corresponding resonance field *μ*_0_*H*_0_, linewidth *μ*_0_Δ*H*, and amplitudes *V_S_* and *V_A_*. The close agreement between the black curves and the raw data demonstrates excellent fitting quality. Based on the parameters obtained from the fit, the symmetric (green line) and anti-symmetric (blue line) Lorentzian peaks are also plotted. Here, the orientation of the symmetric Lorentzian resonance peak (i.e., the sign of *V_S_*) corresponds to the sign of the spin Hall angle of the heavy-metal layer [46].

In principle, Pt possesses a positive spin Hall angle, and the *V_S_* of the device should also present a positive value. In this work, as shown in Figure 4, the current flows through the test device from the negative x-direction, resulting in a negative *V_S_* value in Figure 5. When the magnetic field direction is reversed, the insets in Figure 5a,b indicate that the orientation of the symmetric Lorentzian peak also changes accordingly, which is consistent with the symmetry analysis of the damping-like torque [42,46]. The results exclude the possibility that the signal originates from an unbalanced perpendicular Oersted-field torque. In comparison with Figure 5a,b, it is seen that in the Py_83_Gd_17_ sample, the contribution of the symmetric Lorentzian component is significantly larger, with *V_S_*/*V_A_* reaching 14.47. The symmetric peak nearly coincides with the entire data curve, whereas the *V_S_*/*V_A_* ratio in the pure Py sample is only 0.47. Therefore, it can be anticipated that the strength of the damping-like effective field may be substantially enhanced in the Py_83_Gd_17_ sample. Furthermore, Figure 5c presents the ST-FMR spectra of samples with different Gd contents measured under the same conditions. As the Gd content increases, the overall amplitude of the ST-FMR signal progressively attenuates, which is largely related to the weakening of the AMR effect. In addition, both the position of the resonance peak (*μ*_0_*H*_0_) and the linewidth (*μ*_0_Δ*H*) increase with higher Gd contents and exhibit similar variation trends, as shown in Figure 5d. The resonance field and linewidth show a decrease only in the sample with 20% Gd doping.

**Figure 6:**
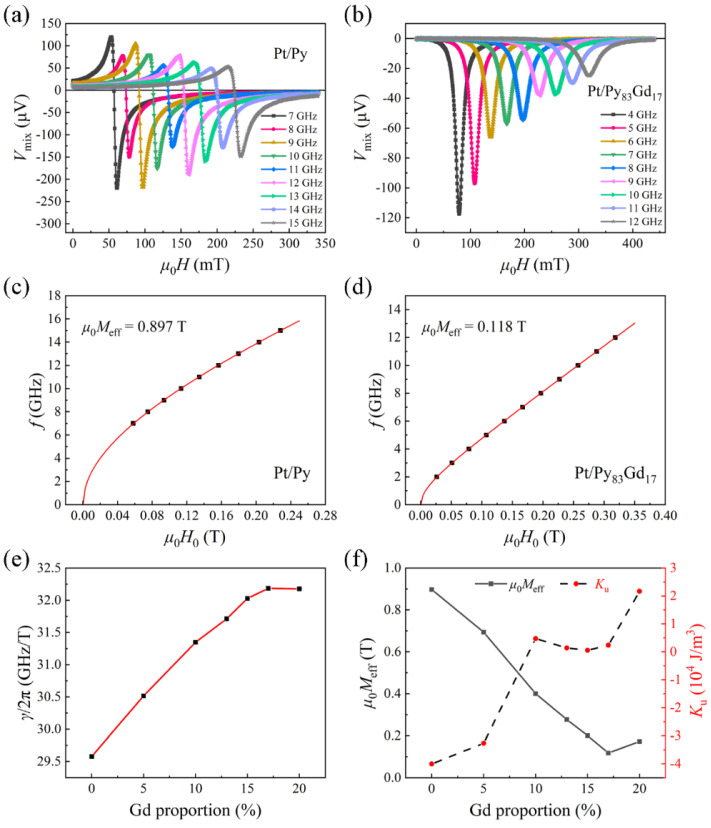
Measurement results of ST-FMR under various frequencies. (**a**,**b**) ST FMR spectra of (**a**) Pt/Py and (**b**) Pt/Py_83_Gd_17_ samples under different frequency ranges with the field at *φ* = 45°. (**c**,**d**) The relationship between frequency f and the resonant field *μ*_0_*H*_0_. (**e**) The variation of *γ*/(2*π*) with the Gd content. (**f**) The variations of *μ*_0_*M_eff_* and *K_u_* as a function of Gd content.

Next, a systematic analysis of the ST-FMR response for each sample at different current frequencies was conducted. Figure 6a,b present the ST-FMR spectra for the Pt/Py and Pt/Py_83_Gd_17_ samples in the frequency ranges of 7~15 GHz and 4~12 GHz, respectively. As the frequency of the RF current increases, the amplitude of the resonance peak generally decreases. In Figure 6a, the variation in the amplitude with frequency does not show a clear monotonic trend, which may be related to the characteristic response of RF cables and connectors or to changes in the RF current power received by the device at different frequencies, thereby affecting the strength of the resonance signal [47]. Since the fitting analysis primarily relies on the symmetry or linewidth of the resonance peak, the amplitude has little influence on the final results. The relationship between the resonance field and frequency for the pure Py sample and the Py_83_Gd_17_ sample is shown in Figure 6c and Figure 6d, respectively. The red curves represent fitted data based on the Kittel equation. The two-parameter fitting based on the gyromagnetic ratio and the effective demagnetizing field (*μ*_0_*M_eff_*) agrees well with the experimental results. Fitting with the Kittel equation further reveals variations in the gyromagnetic ratio of the samples and the in-plane anisotropy of the magnetization. Consequently, during ST-FMR measurements, the deviation between the azimuthal angle of the magnetization and the applied magnetic field is very small. The variation curves of the gyromagnetic ratio and the effective demagnetizing field with Gd content in the samples, obtained from fitting with the Kittel equation, are shown in Figure 6e and Figure 6f, respectively. For the pure Py sample, the fitted gyromagnetic ratio is 29.58 GHz/T. The gyromagnetic ratio is calculated as shown in Equation (3), where g represents the Landé g-factor, and h is the Planck constant.

(3) $$\gamma /(2\pi )=g\mu_{B}/h;$$

Thus, the Landé g-factor for Py in the Pt/Py sample is calculated as *g_Py_* = 2.113, which is consistent with reported results [43,48]. As the Gd content increases, the gyromagnetic ratio also gradually increases, reaching a maximum at x = 17. For the Py_83_Gd_17_ sample, the corresponding gyromagnetic ratio and Landé g-factor are 32.186 GHz/T and 2.30, respectively. As seen in Figure 6e, the variation of the gyromagnetic ratio with composition in Py-Gd alloys is nonlinear, which is related to the difference in gyromagnetic ratios between Py and Gd. Due to its half-filled 4f shell, Gd has zero orbital angular momentum, revealing only a spin contribution. Therefore, the Landé g-factor for Gd is *g_Gd_* = 2, which is not equal to that of Py [49]. When Py and Gd are antiferromagnetically coupled to form an alloy, the variation of the total magnetic moment with composition and the variation of the total angular momentum with composition are not synchronized. Consequently, there exist both an angular momentum compensation point and a magnetization compensation point. From Figure 6e, it can be inferred that the angular momentum compensation point for the Py-Gd alloy lies between 17% and 20%. Since Gd has a smaller gyromagnetic ratio, its angular momentum changes more rapidly with composition. Therefore, when the Gd content is less than or equal than 17%, as the Gd content increases, the total angular momentum decreases faster than the total magnetic moment, leading to a gradual increase in the gyromagnetic ratio of the Py-Gd alloy. After passing the angular momentum compensation point, as the total angular momentum begins to increase again while the total magnetic moment continues to decrease, the effective gyromagnetic ratio of the alloy consequently decreases, which is generally consistent with the trend shown in Figure 6e. However, under such circumstances, the alloy’s gyromagnetic ratio should diverge at the angular momentum compensation point and become negative beyond that point. This phenomenon is not observed in our work. But it should be noted that the theoretically predicted divergence and sign reversal of the gyromagnetic ratio (*γ*) presuppose an ideal angular momentum compensation state with zero net angular momentum in a uniform, infinite medium where magnetic anisotropy effects are negligible. Under these ideal conditions, the precession frequency (and the effective *γ*) is governed by the ratio of the exchange interaction to net angular momentum, leading to a singularity near the compensation point. In real, finite-sized, and inhomogeneous thin-film systems—especially alloys like PyGd—several factors can significantly suppress or completely mask this singular behavior. Also, a previous investigation suggests that the opposing magnetic anisotropy fields of the Py and Gd sublattices must also be considered [49,50]. For instance, the Py-Gd system exhibits a trend in perpendicular magnetic anisotropy (PMA) at higher Gd concentrations. This anisotropy field may provide a finite restoring force for precession, thereby “pinning” the resonance frequency and preventing its divergence at compensation. When the anisotropy fields are taken into account, simulation results at low temperatures show good agreement with the experimental results of this work. In summary, our failure to observe a divergence in *γ* is not because the system is not near angular momentum compensation but because the inevitable magnetic anisotropy and inhomogeneity in real materials compensate for the idealized singularity.

The variation trend in the effective demagnetization field is opposite to that of the gyromagnetic ratio. In Figure 6f, as the Gd content increases, the effective demagnetization field of the samples decreases steadily from 0.897 T to 0.118 T. In the sample with *x* = 20, it rises again to 0.18 T. In ST-FMR measurements, the magnitude of the effective demagnetization field is determined by the combined effects of out-of-plane demagnetization and the perpendicular anisotropy equivalent field, which can be expressed by Equation (4) [51,52].

(4) $$\mu_{0}M_{eff}=N_{z}\mu_{0}M_{s}-\frac{2K_{u}}{M_{s}};$$

In Equation (4), *N_z_* is the demagnetizing factor in the perpendicular direction. For thin-film samples, *N_z_* can be considered as 1. *K_u_* is the perpendicular anisotropy constant, which includes contributions from both interfacial anisotropy and bulk anisotropy.

Therefore, in samples with Gd contents less than or equal to 17%, the reduction in the effective demagnetizing field is closely related to the decrease in saturation magnetization and the enhancement of bulk perpendicular anisotropy. The variation in *K_u_*, calculated according to Equation (4), is shown in Figure 6f. A negative value of *K_u_* indicates the entire in-plane easy magnetization direction of the sample. When the Gd content exceeds 5%, *K_u_* becomes positive, indicating the occurrence of a certain degree of perpendicular anisotropy. For Gd contents between 10% and 17%, perpendicular anisotropy remains relatively weak, with *K_u_* hovering near zero. In contrast, the sample with x = 20 exhibits perpendicular magnetic anisotropy (PMA). This observation is consistent with the conclusions drawn from the earlier M-H curves and MOKE hysteresis loops in Figure 3. However, the increase in the effective demagnetizing field at this point still indicates that the influence of the perpendicular anisotropy equivalent field remains weaker than that of the demagnetizing field. Consequently, when an in-plane magnetic field is applied, the magnetization of the sample can still undergo in-plane ferromagnetic resonance.

### 3.3. Magnetic Damping Results

**Figure 7:**
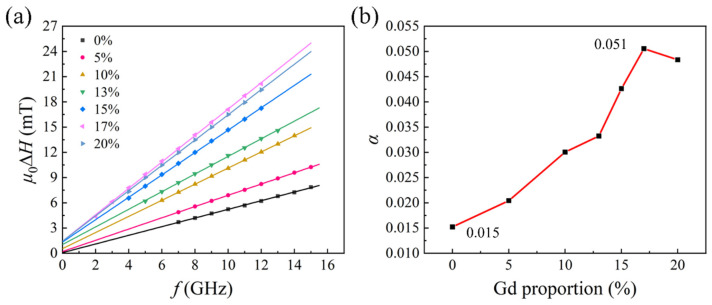
(**a**) The relationship between resonance linewidth and frequency in samples with different Gd contents. (**b**) The variation of the damping coefficient α with the Gd content.

The linewidth of the ST-FMR spectra provides information about damping in the magnetization dynamics process. Therefore, Figure 7a shows the variation in the resonance linewidth with current frequencies for each sample. A good linear relationship between $\mu_{0}\Delta H$ and f can be observed. This linear relationship can be expressed in the form of Equation (5) [53].

(5) $$\mu_{0}\Delta H=W+\frac{2\pi \alpha }{\gamma }f;$$

In Equation (5), *W* represents the linewidth broadening caused by the inhomogeneity of the thin film. When there are inhomogeneities with equal local magnetization in the film, changes in the effective demagnetization field within these inhomogeneous regions cause slight shifts in the resonance relative to the homogeneous regions. The accumulation of these shifts ultimately manifests as a broadening of the resonance peak in the overall ST-FMR spectrum. Clearly, linewidth broadening due to inhomogeneity is independent of frequency and is therefore represented by the linewidth at zero frequency. For an ideal, defect-free, and uniform thin film, the value of *W* is zero. In Figure 7a, the value of *W* is close to zero only when the Gd content is lower than or equal to 5%, indicating that the Py-Gd magnetic layer with low Gd content is still relatively uniform and has few defects. As the Gd content increases further, the lattice structure of Py is gradually disrupted, and the inhomogeneity of the magnetic layer continuously increases, leading to a corresponding increase in the *W* value. On the other hand, two-magnon scattering is also a common mechanism that broadens the ST-FMR spectral linewidth [47,54]. However, two-magnon scattering would cause the linewidth to exhibit a nonlinear dependence on frequency, which is not observed in Figure 7a. Therefore, it is concluded that the contribution of two-magnon scattering to the linewidth can be neglected. The magnetic damping of the samples is a significant factor contributing to the broadening of the ST-FMR spectral linewidth. Also, the slope of the fitted line is related to the Gilbert damping coefficient α. Its variation with x reflects the modulation of spin relaxation mechanisms by Gd doping. According to Equation (5), the Gilbert damping factor (*α*) can be obtained by multiplying the slope of the fitted line in Figure 7a by the corresponding gyromagnetic ratio. The relationship between α and the Gd content in the samples is shown in Figure 7b. For the pure Py sample, the damping factor of Py is approximately 0.015, which is close to the value reported in reference [51]. With the addition of Gd, the damping factor *α* in the Py-Gd magnetic layer shows a large enhancement, reaching a maximum value of 0.051 when x = 17, which is about 2.4 times greater than that of the pure Py sample. It should be noted that the maximum damping parameter obtained at x = 17 does not yet account for the uncertainty introduced by errors in the Gd concentration. This uncertainty may arise from the nominal error in Gd concentration, the standard error in the ST-FMR linewidth fitting, and the spacing of the selected data points. These factors could lead to minor deviations in the position at which the maximum damping parameter occurs. However, it should be made clear that the absence of a quantitative analysis of this uncertainty does not affect the core message that this work ultimately aims to convey to readers. The results allow for the tuning of the magnetic damping within a certain range. The *α* shown in Figure 7b consists of intrinsic and extrinsic damping, which can be expressed as “*α* = *α*_0_ + *α*′”. Intrinsic damping (*α*_0_) primarily originates from relaxation processes caused by electron spin–orbit coupling and other material-specific factors. Previous studies have indicated that Gd doping has a relatively minor effect on tuning the intrinsic damping of Py [4]. Therefore, the extrinsic damping (*α*′) in the Pt/Py-Gd heterostructure resulting from the spin pumping effect may be a key factor [55]. The spin pumping effect refers to the phenomenon in a ferromagnetic/non-magnetic (FM/NM) system where the precession of magnetization in the magnetic layer occurs, and a portion of the spin angular momentum is transferred to the NM layer. In this process, the reduced angular momentum in the magnetic layer subjects its magnetization precession to an additional torque, leading to an increase in its dynamic damping. The extrinsic damping (*α*′) caused by spin pumping can be expressed in the form of Equation (6).

(6) $$\alpha^{\prime }=g\mu_{B}\frac{G_{eff}^{↑↓}}{4\pi M_{s}}\frac{1}{t_{FM}};$$

In Equation (6), $G_{eff}^{↑↓}$ represents the effective spin mixing conductance, and *M_s_* is the saturation magnetization. When the thickness of the Py-Gd magnetic layer is fixed, a smaller *M_s_* leads to a stronger extrinsic damping contribution. In fact, the contribution of the spin pumping effect to the magnetic damping of the system depends on multiple factors, including the spin-mixing conductance, the material and thickness of the non-magnetic layer, the thickness of the magnetic layer, and the FM/NM interface. The present study can only partially corroborate some of these aspects. Therefore, to fully verify the enhancement effect of spin pumping on magnetic damping, additional experiments need to be designed. From Figure 3b and Equation (6), as the Gd content increases, *M_s_* gradually decreases when the Gd content is less than or equal to 17%. Therefore, the extrinsic magnetic damping of the system is enhanced, which is broadly consistent with the variation trend shown in Figure 7b.

**Figure 8:**
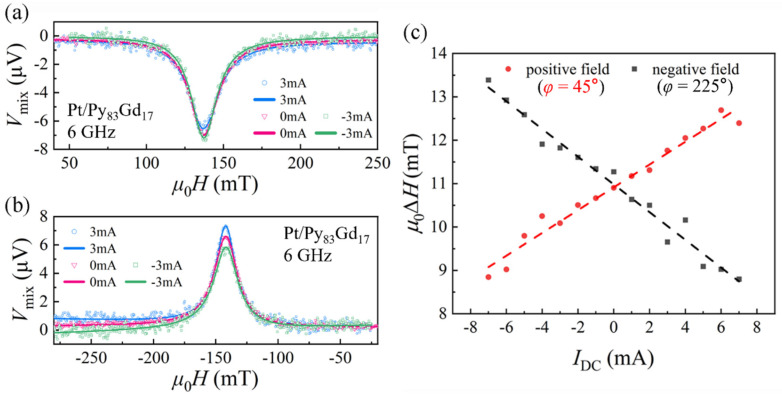
ST-FMR spectra of the Pt/Py_83_Gd_17_ sample under different DC-bias currents *I_DC_* for (**a**) positive (*φ* = 45°) and (**b**) negative (*φ* = 225°) fields with an RF of 6 GHz. (**c**) The variation of linewidth *μ*_0_Δ*H* as a function of *I_DC_*.

The modulation of magnetic damping in the Py-Gd alloy in this study is of critical importance for adjusting the switching speed in RE-based SOT devices. More importantly, this work finds that Gd doping not only increases the linewidth of the ST-FMR spectral line but also enhances the ability of the unit DC current to modulate the linewidth. The injected DC current generates additional spin–orbit torque (SOT) through the spin Hall effect or interfacial Rashba effect. This torque interacts with the magnetic moment dynamics, effectively modifying the Gilbert damping coefficient α. Positive bias injects electron spins partially synchronized with the precession phase, leading to a “negative damping” effect that reduces effective damping and narrows the linewidth. Otherwise, negative bias injects spins misaligned with the precession phase, enhancing energy dissipation and manifesting as “positive damping” that broadens the linewidth. Figure 8 presents the ST-FMR measurement results of the Pt/Py_83_Gd_17_ sample under an applied DC bias current, where the frequency of the RF current used is 6 GHz. For the positive field sweep, the magnetic field is oriented along *φ* = 45°, while for the negative sweep, it is along *φ* = 225°. The corresponding ST-FMR spectra for the forward and reverse sweeps are shown in Figure 8a and Figure 8b, respectively. Clearly, under different DC currents (*I_DC_*), both the linewidth and peak amplitude of the ST-FMR spectra exhibit significant changes, regardless of whether the field is positive or negative. Figure 8c displays the variation of the resonance linewidth with the bias current. A relatively good linear relationship between *μ*_0_Δ*H* and *I_DC_* can be observed. Under the positive magnetic field, as the bias current *I_DC_* increases, the ST-FMR linewidth (*μ*_0_Δ*H*) becomes larger, and the absolute value of the resonance peak amplitude decreases, which corresponds to the increase in the effective magnetic damping of the Py-Gd alloy compared to the pure Py sample. Here, the slope between *μ*_0_Δ*H* and *I_DC_* is defined as η. During the positive field sweep, *η* is positive. When the magnetic field direction is reversed, *η* changes from positive to negative. In this case, increasing the bias current *I_DC_* reduces the linewidth, while the resonance peak amplitude increases. This observation also allows us to rule out the possibility of linewidth changes caused by thermal effects. Through linear fitting, the average absolute slope of the linewidth *μ*_0_Δ*H* versus *I_DC_* in the Pt/Py_83_Gd_17_ sample is found to be 0.291 mT/mA. Compared to the result of *η* = 0.0263 mT/mA in the Pt/Py sample, Gd doping enhances the ability of the unit DC current to modulate the linewidth by approximately 10 times. This is of significant importance for spin-torque nano-oscillators. Linewidth modulation is required to achieve stable magnetization precession, as it can substantially reduce the driving current density needed for microwave emission, thereby lowering device power consumption and decreasing the probability of device breakdown.

### 3.4. The Analysis of Spin Hall Angle

In the end, the spin Hall angle (*θ_SH_*) of Pt/Py-Gd bilayers is studied using ST-FMR. In this work, *θ_SH_* is calculated by line-shape analysis. Thus, *V_S_* and *V_A_* in Equation (2) can be further expressed as

(7) $$V_{S}=V_{0}\frac{1}{\alpha (2\mu_{0}H+\mu_{0}M_{eff})}\mu_{0}H_{DL};$$

(8) $$V_{A}=V_{0}\frac{\left(\mu_{0}H_{FL}+\mu_{0}H_{Oe}\right)}{\alpha \left(2\mu_{0}H+\mu_{0}M_{eff}\right)}\sqrt{\left(1+\frac{\mu_{0}M_{eff}}{\mu_{0}H_{0}}\right)};$$

Here, in Equations (7) and (8), *H_DL_* and *H_FL_* refer to the damping-like SOT field and field-like SOT field, respectively. *H_Oe_* is the current-induced Oersted field. Equations (9) and (10) present the expression of *H_DL_* and *H_Oe_*, respectively. Generally, *θ_SH_* is closely related to the damping-like SOT field [10,56,57]. Therefore, the spin Hall angle in this work can be calculated using Equation (11).

(9) $$H_{DL}=\hbar \theta_{SH}J/2e\mu_{0}M_{s}t_{FM};$$

(10) $$H_{Oe}=Jt_{HM}/2;$$

(11) $$\theta_{SH}=\frac{V_{S}}{V_{A}}\frac{e\mu_{0}M_{s}t_{FM}t_{HM}}{\hbar }\sqrt{\left(1+\frac{\mu_{0}M_{eff}}{\mu_{0}H_{0}}\right)};$$

**Figure 9:**
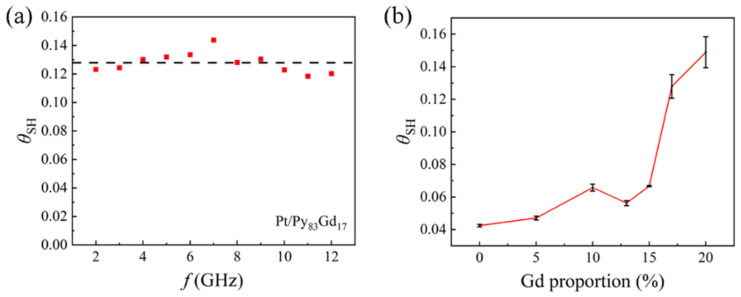
Spin Hall angle *θ_SH_* obtained by line-shape analysis. (**a**) Spin Hall angle of Pt/Py_83_Gd_17_ sample at different frequencies. (**b**) Spin Hall angle in samples as a function of different Gd content.

In Equation (11), *e* and *ħ* represent the elementary charge and the reduced Planck constant, respectively; *t_HM_* and *t_FM_* denote the thicknesses of the heavy-metal layer and the ferromagnetic layer, respectively; *J* is the current density flowing through the heavy-metal layer. Based on Equation (11) and ST-FMR results, the spin Hall angle of the Pt/Py-Gd bilayers can be acquired. Figure 9a represents the spin Hall angle of the Pt/Py_83_Gd_17_ sample as a function of frequency. It is noted that results fluctuate around 0.128 under different frequencies. The variation in the spin Hall angle for samples with different Gd contents is shown in Figure 9b, where the error bars represent the statistical variation of the *θ_SH_* values measured at different frequencies. The *θ_SH_* for the pure Py sample is about 0.042. Based on previously reported results, the spin Hall angle of Pt lies between approximately 0.06 and 0.07, which is a little smaller than the conventional value [10,58]. Published studies have shown that the spin Hall angle of a system can be influenced by multiple sources of spin currents. For example, a Ta/W composite heavy-metal layer can enhance the spin Hall angle of the system, since both Ta and W possess a negative spin Hall angle [59]. Similar observations have been confirmed in spin–orbit torque (SOT) systems containing rare-earth elements. In Pt/Co/Ho magnetic multilayers, the spin Hall angle of the system is weakened because Ho and Pt share the same sign of the spin Hall angle [33]. Similarly, in this work, the spin Hall angle of Pt/Py systems is lower than that of pure Pt, leading to the reasonable consideration that the spin Hall angle of Py is likely negative. With the addition of Gd, *θ_SH_* increases monotonously. In particular, there is a sharp rise in *θ_SH_* when the Gd content reaches 17%. The *θ_SH_* reaches its maximum value of about 0.149 in the Pt/Py_80_Gd_20_ sample, which is roughly 2.5 times larger than that of the pure Py sample. Compared with typical SOT current–spin conversion materials like Pt, Ta, W, and Hf, the spin Hall angle of Py-Gd in this work is highly competitive [10,58,60,61]. It should be made clear that, without conducting further experiments, the effect of Gd concentrations on the spin Hall angle for x > 20 remains unknown. But there is no universal trend regarding the doping concentration at which the spin Hall angle reaches its maximum in rare-earth-containing alloy systems based on published studies [32,35]. In addition, we need to acknowledge that the enhancement of the spin Hall angle may involve both bulk and interface effects [59]. And it is very challenging to completely distinguish these two effects in our system. But based on the existing data, we still believe that the bulk effect dominates the enhancement in the Pt/PyGd system. In our work, the Gd doping concentration is systematically varied, and the observed changes in damping parameter α and SOT efficiency show continuous and significant trends, which are more consistent with a gradual change in bulk material properties. In addition, the Pt layer thickness in our work is fixed, and the main interface of Pt/Py-Gd remains unchanged. Therefore, changes in interface effects should primarily stem from the altered properties of the Py-Gd layer itself, which can itself be viewed as the manifestation of the doping-induced bulk effect at the interface. In all, the findings of this work further confirm the significant role of alloys and rare-earth elements in SOT studies, demonstrating that employing Py-Gd alloys is an effective route for fabricating next-generation spintronic devices with high charge-to-spin conversion efficiency.

## 4. Conclusions

In this work, Pt/Py_100−x_Gd_x_ bilayers were prepared using co-sputtering technology. The ST-FMR technique was employed to investigate the magnetic damping properties and the variation in the spin Hall angle with Gd composition. By controlling the composition of the Py-Gd alloy, the effective gyromagnetic ratio and the magnetic damping coefficient of the system can be effectively tuned. When *x* is less than or equal to 20, both the gyromagnetic ratio *γ*/(2*π*) and the magnetic damping coefficient α of Py_100−x_Gd_x_ increase significantly with higher Gd content, reaching their maximum values when x = 17. The maximum *α* is 0.051, which represents an approximate 2.4-fold increase compared to the pure Py sample. The relationship between Gd compositions and the spin Hall angle was analyzed using the ST-FMR technique. The results indicate that the spin Hall angle in the Pt/Py-Gd system increases monotonically with higher Gd content, reaching a maximum of 0.149. This value shows a significant improvement compared to conventional single-element heavy metals used for spin–orbit torque. We admitted that the extraction of the spin Hall angle in this work relies on simplified models and does not fully account for interfacial spin memory loss and spin diffusion effects in multilayer structures. However, the existing data still supports the conclusion that the enhancement of the system’s spin Hall angle is primarily dominated by bulk effects. Additionally, the behavior of the observed trends in damping and spin Hall angle with Gd concentration beyond the concentration range studied here remains unclear and requires further experimental verification. We emphasize that these limitations do not invalidate the core conclusions but instead point toward directions for future research. Meanwhile, Gd doping substantially enhances the ability to modulate the ferromagnetic resonance linewidth. For instance, in the Pt/Py_83_Gd_17_ sample, the linewidth change per unit current is increased by approximately 10 times compared to the Pt/Py sample. The systematic investigation of magnetic damping and spin Hall angles in Py-Gd alloys presented in this work contributes to modulating the magnetization switching speed in spintronic devices and designing devices with high charge-to-spin conversion efficiency. Our study reveals that rare-earth (Gd) doping enables a significant enhancement of spin–orbit torque strength while maintaining relatively low damping, which provides a new pathway for the co-optimization of damping and torque efficiency. It should be noted that this approach is not limited to the Py-Gd system and may be extended to other rare-earth-transition-metal alloys, enabling the development of novel spin injection sources, high-frequency spin nano-oscillators, and ultrafast magnetization switching devices.

## Data Availability

The raw data supporting the conclusions of this article will be made available by the authors on request.
